# The interaction of a self-assembled nanoparticle and a lipid membrane: Binding, disassembly and re-distribution

**DOI:** 10.1016/j.heliyon.2024.e39681

**Published:** 2024-10-23

**Authors:** Lilia Milanesi, Salvador Tomas

**Affiliations:** Department of Chemistry, University of the Balearic Islands, Ctra. Valldemossa, Km 7.5. 07122, Palma de Mallorca, Spain

## Abstract

Here we report a detailed study of the interactions of nanoparticles, formed by the self-assembly of cholesterol-containing porphyrins, with lipid membranes. We show that the interaction is a two-step process: first, the docking and fusion, then, the redistribution of the building blocks of the self-assembled nanoparticles (SANs henceforth). Analysis of the binding and structural data is consistent with the docking step being driven by a multivalence cooperative effect and with the formation of SAN aggregates on the membrane, whilst the solubility of the cholesterol anchor in the membrane is key to both the fusion and redistribution of the SANs building blocks. The tendency of the SAN to aggregate in the membrane helps explain the photosensitizer properties of the SANs, essential to their anti-microbial activity. The solubility of the cholesteryl anchors drives fusion to the membrane and de-assembly of the SAN, explaining the capability of the SANs to deliver therapeutic cargos at the lipid interface. The subsequent redistribution of the SANs building blocks offer a plausible pathway to body clearance that is not immediately available to hard nanoparticles. These properties, and the modularity of the synthesis, point to the SANs being an excellent platform for the development of nanomedicines. An unexpected consequence of unraveling the mechanism of membrane interaction of these SANs is that it allows us to derive a value of the free energy of binding of cholesterol (the membrane anchor of the SAN building blocks) to a lipid membrane, that is consistent with the literature values. This is an additional property that can be exploited to determine the affinity of a variety of membrane anchors to membranes of various compositions.

## Highlights

• Porphyrin-cholesterol self-assembled nanoparticles bind to and fuse with lipid membranes.
• Cholesterol membrane solubility is key to fusion and disassembly of the nanoparticle.
• Multivalence is key to the efficient membrane binding of the nanoparticle.
• Analysis of the data allows estimating the binding energy of the cholesterol anchor.

## Graphical abstract

**Figure:**
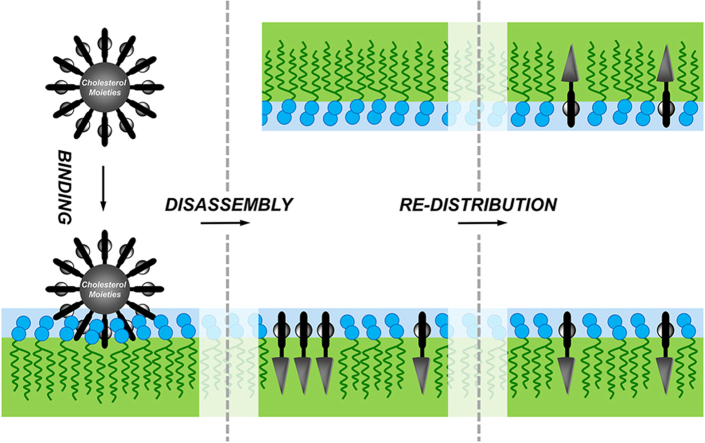
Self-assembled nanoparticles are disassembled on a lipid membrane and the building blocks distributed amongst all the available membranes.

## 1 Introduction

Nanotechnology is now commonly applied across various industries, including the medical field [1,2]. It typically involves the use of nanoparticles, making it crucial to understand how these particles interact with living organisms. This knowledge is essential for assessing potential toxic effects derived from industrial uses [3,4] and for developing more effective nanoparticle-based materials for nanomedicine and biomedical research [5,6]. Nanoparticles used in these applications come in diverse structures and compositions. Inorganic nanoparticles, such as metal nanoparticles and quantum dots, are primarily used in live cell imaging and for medical diagnostics [[7], [8], [9]]. Bio-organic nanoparticles, including nanogels [10,11], protein nanoparticles [12,13], lipid nanoparticles [14] and lipid vesicles [15,16], are often employed as drug carriers. However, common challenges include poor control over the distribution of these carriers within the body [17] and concerns about the clearance and potential toxic accumulation of the non-biological carrier components [[18], [19], [20], [21]].

**Fig. 1:**
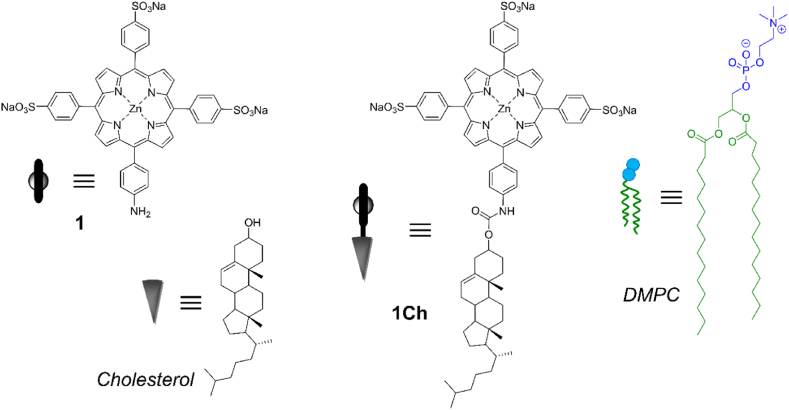
a) Chemical structure of **1**, **1Ch**, DMPC and cholesterol and the carton representations used in this work.

**1Ch** (Fig. 1) is an amphiphilic molecule composed of a tri-anionic porphyrin head group and a cholesterol tail. In water or aqueous buffers, **1Ch** assembles into micelles of unusually large stability (CMC 11 nM) which has prompted us to use the term self-assembled nanoparticles (SANs) to describe them [22]. In these nanoparticles, the porphyrin head group, containing a Zn metal centre, binds to ligands bearing basic nitrogen atoms with affinities that are enhanced 10–100 fold in relation to their binding to water soluble porphyrin **1**. This enhancement has been attributed to the synergic action of the metal coordination and the hydrophobic effect, at play within the inter-porphyrin ring cavities on the surface of the nanoparticle and, for multivalent ligands, to a multivalent effect [22]. A version of these SANs, containing Co rather than Zn at the metalloporphirin head-group was loaded with an anti-mycobacteria pro-drug. We demonstrated that these Co-metalloporphyrin based SANs are efficient drug carriers and photosensitizer against a model of mycobacteria [23]. The introduction of Co rather than Zn in the porphyrin moiety demonstrates that the formation of the nanoparticle is not hampered by the presence of different metals at the center of the porphyrin ring. The SAN also tolerates the loading of small-molecule drugs. Taken together, these properties open up the possibility of generating SANs of a various compositions as drug carriers.

Other than assembling into nanoparticles, the SAN building block, amphiphile **1Ch**, can be incorporated into lipid membranes. Once incorporated, it can be used as a minimal model of a membrane-anchored receptor. When anchored in a membrane **1Ch** can be found in a fully dispersed monomeric form, favored at low in-membrane concentrations, or in the form of clusters [[24], [25], [26]]. The nature of these clusters and their mechanism of formation depends on the physical state of the membrane **1Ch** is anchored on. For membranes composed of a single, pure phospholipid, above the phase transition temperature (e.g., in the liquid disordered phase, L_α_) the clustering tendency is negligible, while below the transition phase temperature (e.g., gel phase, L_β_) the clustering follows a cooperative nucleation-growth mechanism, akin to phase separation, leading to the formation of larger clusters [26]. For membranes where the liquid ordered phase, L_o_, is present (e.g., with a mol fraction of cholesterol of 0.2 at 30 °C [27]), the clustering follows an isodesmic, non-cooperative, mechanism, leading to small clusters composed of few molecules [24,25].

Lipid membranes containing membrane-anchored **1Ch** are generated by mixing ethanolic solutions of lipids and **1Ch** to the desired proportions, co-evaporating the organic solvent and suspending the mixture into the aqueous buffer. It is therefore reasonable to infer that there is a mechanism by which **1Ch** can be incorporated into a lipid membrane directly from a SAN. Elucidating this mechanism will shed clues on the interactions of these nanoparticles with biological membranes. This knowledge is a key requirement to fully exploit this class of SANs as nano-carriers for the development of drug delivery systems.

In the present work we set out to study the mechanism of membrane-SAN interactions in detail using a minimal membrane system composed of lipid vesicles, taking advantage of the favorable spectroscopic properties of the porphyrin moiety, which allow us to study in detail the process by monitoring changes in the UV–Vis spectrum. The study reveals details of the nanoparticle-membrane interaction that helps understanding how they may interact with living cells. In particular, we observe that the initial docking is driven by a multivalence cooperative effect and that, in common with other nanoparticles, they tend to form aggregates onto the membrane [28]. We observe that the SAN fuse to, and disassemble in, the membrane, and later re-distribute amongst lipid vesicles, a process driven by the solubility of the cholesterol anchor in the membrane. This mechanistic insight helps understanding the role of the cholesterol moiety in making the SAN an efficient delivery platform of a prodrug against mycobacteria in our earlier work [23]. It is worth noting that cholesterol is a key component of other self-assembled, soft nanoparticles such as solid lipid nanoparticles, extensively developed for drug delivery and transfection applications [29,30].

## 2 Results and discussion

**Fig. 2:**
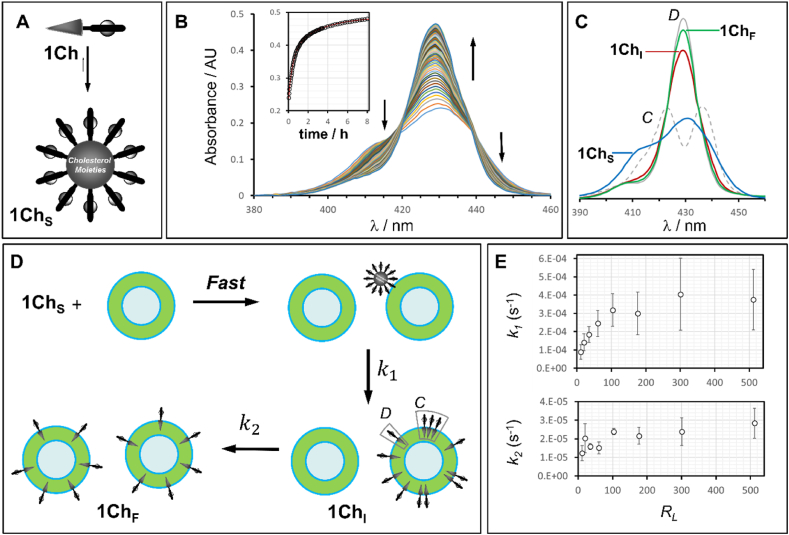
A. Schematic representation of the formation of SAN of **1Ch**. B. Changes in the UV spectrum upon addition of **1Ch**_**S**_ to vesicles composed of DMPC and Cholesterol in aqueous buffer. The concentration of **1Ch** was 1.5 μM and the total concentration of lipids was 450 μM. The contribution of the vesicle scattering to the UV spectrum has been removed (see “Data Analysis” in the Supplementary Information for details on data processing). The inset show the changes of the absorbance at 429 nm with time (empty circles) and fitting to a two steps kinetic process (continuous red line). C. Soret band of the **1Ch**_**s**_ species, recorded before addition of lipid vesicles (blue trace) and of **1Ch**_**I**_ (red trace) and **1Ch**_**F**_ (green trace) species, extrapolated from the fitting of the data. The hypothetical Soret band of pure membrane clustered and dispersed forms (*C* and *D* respectively, dashed and continuous grey lines, see Ref. [24]) are also shown for comparison. D. Cartoon representation of the proposed mechanism of incorporation of **1Ch** into the membrane of lipid vesicles (*C* and *D* indicates the clustered and dispersed forms of membrane-embedded **1Ch**). E. Changes in the rate constants with the ratio of concentration of lipid over the concentration of **1Ch**, *R*_*L*_. The error bars represent twice the standard deviation of two measurements.

Amphihilic Zn metaloporphyrin **1Ch** embeds efficiently into lipid membranes composed of DMPC or DMPC/Chol in a 8:2 M ratio (Fig. 1). In earlier work, we have seen that, when anchored in membranes in the liquid disordered state, L_α_ (e.g., pure DMPC at temperatures above the phase transition temperature) **1Ch** shows a very little tendency to form clusters [26]. However, when a liquid ordered phase L_o_ is present (e.g., for membranes composed of DMPC/Chol in a 8:2 M ratio at 30°C), a weak clustering of **1Ch** is revealed by characteristic changes in the UV spectrum [24,25]. The UV spectrum shows sharp bands for monomeric **1Ch**, dominant at low membrane loading (at or below 1 %) to increasingly broad bands, consistent with the presence of porphyrin H-aggregates, when the loading in the membrane is increased above 1 % [24]. Large concentrations of **1Ch**, which bears a cholesteryl moiety as a membrane anchor, may contribute to increase the amount of L_o_ phase domains in the membrane, thus impacting the clustering of **1Ch**. However, regardless of what the precise contributing factors to the clustering of **1Ch** in the membrane are (i.e., recruiting within L_o_ domains of **1Ch** or porphyrin-porphyrin interactions), the changes in the UV data are consistent with an isodesmic, non-cooperative aggregation mechanism. From the analysis of the data, the equilibrium constant between the clustered *C* and dispersed *D* forms, *K*_*DC*_, at 30 °C was determined and the UV spectrum of the *C* and *D* forms of **1Ch** was extrapolated (Fig. 2C) [24]. We also found that **1Ch** incorporates efficiently into the lipid membranes up to a loading of 8 % molar which is the saturation point of the membrane, e.g., the presence of additional **1Ch** leads to the co-formation of nanoparticles of **1Ch** [24].

With a CMC of 11 nM [22], the addition of pure **1Ch** to water results in the near quantitative assembly into nanoparticles. For example, in our typical experimental conditions, (**1Ch** 1.5 μM) more than 99 % of **1Ch** is found as component of a SAN (Fig. 2A). The SAN show a UV spectrum that is distinct to that of in-membrane clustered **1Ch** (Fig. 2C) [22,24]. In line with the setup used in our previous work, the vesicles used in here are composed of a mixture DMPC/Cholesterol 8:2 and the experiments carried out at 30 °C, so the previously characterized clustering of **1Ch** can be used as an indirect measure of the in-membrane concentration of **1Ch** [24].

When **1Ch** SAN are added to pre-formed lipid vesicles, their characteristic UV spectrum immediately begins to change, consistent with their disassembly as **1Ch** monomers into the membrane of the vesicles (Fig. 2B). Recording of the UV spectra for longer times (up to 8 h) shows that, following an initial, comparatively rapid change (e.g., completed in approximately 1 h), the Soret band of the UV spectra of the porphyrin moiety continue to sharpen (Fig. 2B). In an initial data analysis, the UV spectral changes were fitted to a kinetic model that assumes the overall process occurs in two irreversible stages, each being first order with respect to the concentration of **1Ch** molecules. Specifically, we assume that **1Ch**, as a component of a SAN (referred to as **1Ch**_**S**_), first transforms into an intermediate state (**1Ch**_**I**_), which then undergoes a subsequent transformation to the final state (**1Ch**_**F**_) (Fig. 2D). The experiments were carried out by mixing **1Ch** at constant concentration with increasing concentrations of lipid vesicles, aiming for vesicles with a loading of **1Ch** ranging from 0.2 to 8 % molar membrane composition upon **1Ch** incorporation onto the vesicle. The fit of the data to the model is excellent in all cases (Fig. 2B, Supplementary Fig. S1). The apparent rate constants for both the first and the second stages is independent of the lipid concentration for all samples in which the vesicle loading after **1Ch** incorporation is around or lower than 1 % (that is, the molar ratio of lipid over **1Ch**, termed *R*_*L*_, is around or larger than 100). For higher loadings (that is, lower values of *R*_*L*_), the rate constant of the first stage (i.e. associated with the formation of the intermediate state **1Ch**_**I**_), *k*_*1*_, decreases, but for the second stage, the formation of **1Ch**_**F**_, changes in the constant *k*_*2*_ are less significant (Fig. 2E–Supplementary Table 1).

The fitting of the data allows us to extrapolate the UV spectrum of hypothetical pure species **1Ch**_**I**_ and **1Ch**_**F**_ (Fig. 2C, Supplementary Fig. 1S). The spectra exhibit characteristics that are a combination of the in-membrane clustered form (*C*) and the monomeric, dispersed form (*D*), with variations depending on the **1Ch** loading on the membranes. In all instances, the spectrum of **1Ch**_**I**_ shows a greater relative contribution of the clustered form *C*, pointing to a mechanism where **1Ch** becomes diluted within the membrane as it transitions from **1Ch**_**I**_ to **1Ch**_**F**_.

Overall, these results support the following mechanism:

(i) Irreversible SAN-Membrane docking. This step takes place on a faster timescale than the experiment, as indicated by the rate constants being largely independent of lipid concentration. Additionally, the UV spectrum of the intermediate membrane-embedded form (**1Ch**_**I**_) is broader than that of the final form (**1Ch**_**F**_) (Fig. 2C, Supplementary Fig. 1S). The spectrum reveals a larger contribution of the *C* form, consistent with a higher loading of **1Ch** in the membrane at the intermediate state (**1Ch**_**I**_) than at the final state (**1Ch**_**F**_). The implication is that, initially, not all lipid vesicles bind with SANs, resulting in a larger loading in those vesicles that do bind to SANs. Reversible docking would allow for the redistribution of SANs among all vesicles before **1Ch** is incorporated into the membrane, thus this scenario implies that SAN-membrane docking is irreversible.
(ii) Slow SAN-membrane fusion. During this stage, **1Ch** molecules diffuse from the SAN to the membrane, characterized by the rate constant *k*_*1*_, leading to the intermediate state **1Ch**_**I**_**.** The dependence of *k*_*1*_ at very high **1Ch** percentages in the membrane (e.g., above 1 %, *R*_*L*_ below 100) may reflect changes in the membrane's properties at high **1Ch** loadings. In this scenario, the presence of the clustered form *C* and, plausibly, the increase of the L_o_ phase in the membrane [27], alters the membrane's ability to extract **1Ch** from the membrane-bound SAN. Further consideration of this possibility is discussed below.
(iii) Slower redistribution process. In this final stage, embedded **1Ch** redistributes from vesicles with high loading to those with low loading, governed by the rate constant *k*_*2*_, resulting in the final state, **1Ch**_**F**_**.** The slight decrease of *k*_*2*_ at high loadings may also be indicative of the role played by the presence of *C* and the increase of the L_o_ phase during this stage. To validate this mechanism, we conducted a detailed analysis of each step.

### 2.1 Irreversible docking

**Fig. 3:**
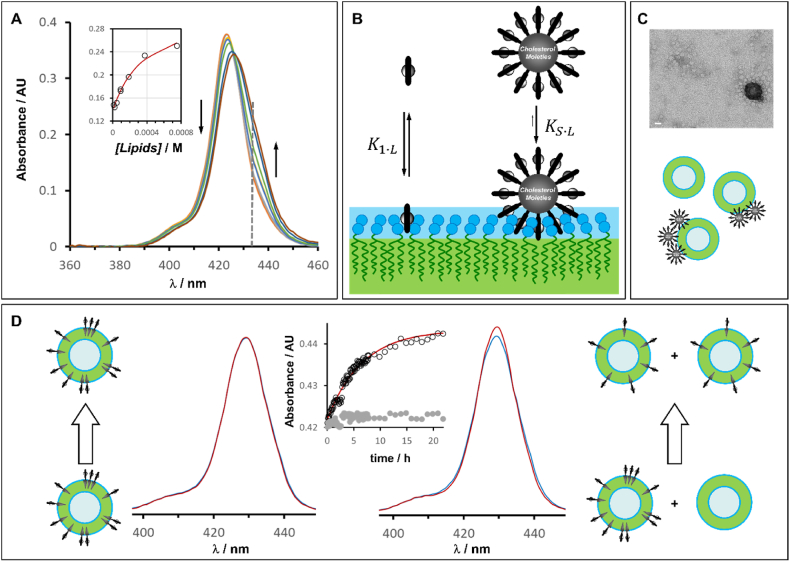
A. Changes in the Soret band of the UV spectrum of **1** (1 μM in buffer) upon addition of increasing amounts of lipid vesicles. The inset shows the changes in absorbance at 434 nm (marked in the main figure by a grey dotted line) as empty circles. The fit to a 1 to 1 binding model is shown as a red continuous line. B. Cartoon representation of the binding of **1** to the interface of a lipid membrane and the corresponding multi-valent binding of **1Ch**_**S**_ to the membrane. C. Top panel: Transmission Electron Micrograph (negative stain) of a sample of **1Ch**_**S**_ in buffer. It can be appreciated how **1Ch**_**S**_ aggregates around stain particles. The scale bar represents 10 nm. Bottom panel: cartoon representation of the non-homogeneous distribution of **1Ch**_**S**_ amongst vesicles. D. Left side: Soret band of the UV spectrum of membrane-embedded **1Ch** from a sample of vesicles formed by co-evaporation of **1Ch** and lipids stock solutions ([**1Ch**] = 1.5 μM, ratio Lipids/**1Ch** = 50), blue trace. The red trace is the Soret band of the same sample after 22 h at room temperature. The cartoon represents the absence of redistribution, consistent with the behavior of the spectra. The inset at the center shows the evolution of the absorbance at 429 nm during this period (full grey circles). Right side: Soret band of an aliquot of the same sample (blue trace) and 22 h after addition of blank vesicles over the sample, bringing the ratio lipids/**1Ch** up to 500 (red trace). The cartoon to the right is the idealized representation of the redistribution of **1Ch** amongst all available membrane. The inset at the center shows the changes in absorbance at 429 nm during this period (empty circles) and the best fit to a first order kinetic (red trace).

The irreversible docking of the SAN suggests a tight binding to the vesicle membrane. To obtain an estimate of the affinity of our SAN for the lipid membrane, we used water soluble porphyrin **1** as a model of **1Ch** head group. We measured the affinity of **1** for the vesicle membrane using UV titration methods. By fitting the data to a 1:1 binding model, we obtained an apparent bimolecular binding constant for the **1**·Lipid complex, *K*_*1·L*_, of 2800 M⁻^1^ (Fig. 3A. For a detailed explanation of the binding model used, see the “Data Analysis” section in the Supplementary Information). In absence of a ligand that binds to the metal center of **1** at the membrane interface, the driving force for the binding of **1** is attributed to the hydrophobic effect. The SAN surface features multiple porphyrin moieties similar to **1**, which are known to prefer hydrophobic ligands [22]. Therefore, it is reasonable to infer that the lipids in the membrane behave as (relatively) hydrophobic ligands for the inter-porphyrin cavities of the SAN, with the membrane itself acting as a multivalent ligand. Given that the SAN has numerous binding sites, it behaves as a multivalent receptor for the membrane, leading to a multivalent cooperativity effect (Fig. 3B). The apparent binding affinity constant, *K*_*S·L*_, of a multivalent receptor (here our SAN, bearing multiple lipid-binding inter-porphyrin cavities) for a multivalent ligand (here the membrane, bearing multiple lipid molecules) can be approximately calculated as:

(1) $$K_{S∙L}={K_{1∙L}}^{n}{EM}^{n-1}$$

where *EM* is the effective molarity, a measure of the mutual relative concentration of the binding sites. Typical values of *EM* in molecular recognition events are found in the range 0.1–10 M, the latter representing a high limit value where mid-size molecules are in close contact with each other in solution [31]. In the SAN-membrane binding interface, there is a close proximity of the mutual binding sites that make it reasonable to attribute a higher end value to the *EM*. For simplicity of calculations, we assume that *EM* is 1 M. Using this value, we have that as little as 3 binding sites acting simultaneously will yield an approximate binding constant of 2 × 10^10^ M^−1^. Given the inherent flexibility of a membrane where the liquid disordered phase L_α_ coexist with liquid ordered phase L_o_ [27], such as ours, it is very likely that many more than 3 binding sites can contribute to the formation of the SAN-membrane binding interface, which will lead to a tight docking of the SAN into the membrane. While tight binding to the membrane alone facilitates an irreversible process, it doesn't necessarily prevent SAN dissociation. However, our UV data indicate that as soon as docking occurs, **1Ch** molecules begin disassembling from the SAN, diffusing into the membrane as a mixture of dispersed and clustered forms, *D* and *C*. This process renders the SAN binding irreversible.

### 2.2 Initial distribution of membrane embedded **1Ch**

The process of tight and irreversible docking of nanoparticles onto vesicles, where each docking event occurs independently, should result in a Poisson distribution of SAN among the vesicles [32]. By using the Poisson distribution, the apparent loading can be calculated if the ratio of SAN to vesicles in a given sample is known. The number of vesicles present can be determined based on the average size of the produced vesicles, the area occupied by each lipid molecule, and the concentration of lipids. Similarly, the number of SANs can be estimated from the nanoparticle size, the total concentration of **1Ch**, and the area each **1Ch** molecule occupies. Detailed calculations of these parameters, derived from experimental measurements of both vesicle and nanoparticle sizes, as well as our analysis of the Poisson distribution, are presented in the “Poisson Distribution” section of the Supplementary Information [33,34].

In our experiments, the **1Ch**_**I**_ spectrum is attributed to an intermediate state of **1Ch** embedded within the lipid membranes, following the diffusion of **1Ch** molecules into the membrane after initial docking. The Poisson distribution analysis suggests that the distribution at the intermediate stage should resemble in fact a normal distribution, with minimal changes taking place after reaching the **1Ch**_**I**_ stage. However, comparing the UV spectra of **1Ch**_**I**_ and **1Ch**_**F**_ with those of the *C* and *D* forms, it can be inferred that the loading of **1Ch** at the intermediate state (**1Ch**_**I**_) is 2–3 times larger than at the final state (**1Ch**_**F**_) (Fig. 2C and Supplementary Fig. S4D). This finding supports a scenario where SAN docking preferentially occurs on vesicles that already contain nanoparticles, rather than in a completely independent manner. The tendency of nanoparticles to form aggregates when binding to lipid membranes has been described and attributed to changes in the structure of the membrane interface following the initial nanoparticle docking [28]. In the case of **1Ch** SANs, a tendency to form aggregates when deposited into surfaces is also observed in TEM samples. (Fig. 3C, Supplementary Fig. S4).

### 2.3 **1Ch** re-distribution

The transition between the **1Ch**_**I**_ and **1Ch**_**F**_ is consistent with the migration of **1Ch** between vesicles. To test this hypothesis, we conducted an experiment where vesicles were initially produced with **1Ch** incorporated into their membranes through the co-evaporation of ethanolic stock solutions of lipids and **1Ch** (details provided in the experimental section of the Supplementary Information). These **1Ch**-loaded vesicles were then brought into contact with vesicles made of pure lipids. UV monitoring of the pre-loaded vesicle samples showed no significant change in the UV spectrum over a 22-h period. However, when this sample was exposed to **1Ch**-free lipid vesicles, the spectrum became sharper, indicating the dilution of **1Ch** among the available vesicles (Fig. 3D). The rate constant for this process was determined to be 3.1 × 10^−5^ ± 8.0 × 10^−6^ s^−1^, (error quoted as twice the standard deviation of 2 measures) which is consistent, within the margin of error, with the rate constant *k*_*2*_ attributed to the conversion of **1Ch**_**I**_ to **1Ch**_**F**_ in experiments where SANs are added to vesicles (Supplementary Table 1S and Fig. 2E). An alternative mechanism for in-membrane dilution of **1Ch** would be the translocation, or flip-flop motion, of **1Ch** within the two leaflets of the same membrane. Indeed, the reported rate of cholesterol flip-flop is in the same order of magnitude of *k*_*2*_ [35]. However, the translocation of **1Ch** would involve not only the translocation of the cholesterol moiety, but also the permeation, through the membrane, of the polyanionic porphyrin head-group. In our earlier work, we have found that porphyrins similar to **1** are membrane impermeable for periods of up to 48 h [36]. In light of these precedents, a contribution of **1Ch** flip-flop to the in-membrane dilution phenomenon is deemed unlikely in the time-scale of the experiments performed here.

### 2.4 Kinetic model and transformation pathway of a SAN into membrane-embedded **1Ch**

As described above, the overall changes observed in the UV spectrum are consistent with a model that involves two consecutive first-order events, both dependent on the concentration of **1Ch**, following the rapid docking of SAN onto the membranes. In the initial stage, UV changes are attributed to the disaggregation of the nanoparticle's building blocks within the membrane. This process is similar to the dissolution of a solid **1Ch** particle (the SAN) into the lipid vesicle, where the membrane acts as the solvent. For samples with loadings at or below 1 % (or a lipid-to-**1Ch** ratio, *R*_*L*_, equal or greater than 100), the rate constant for this initial process, *k*_*1*_, remains constant within the margin of error and can be equated to the rate of **1Ch** dissolution into the membrane, as described by the Noyes-Whitney solution model [37]. However, with higher loadings (e.g., up to 8 % or an *R*_*L*_ of 11), *k*_*1*_ decreases (Fig. 2E, Supplementary Table S1).

**Fig. 4:**
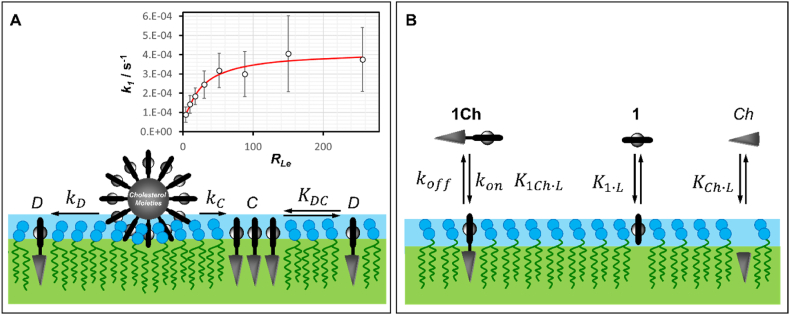
A. Cartoon representation of the dissolution of **1Ch** onto the membrane following SAN membrane docking. The equilibrium between the clustered (*C*) and dispersed (*D*) forms of **1Ch** is also shown. The inset shows the changes of *k*_*1*_ (values from Supplementary Table S1) with the effective ratio lipids over **1Ch** (empty circles) and the best fitting to equation (3) (red trace). B. Cartoon representation of the membrane binding equilibria of **1Ch**, **1** and the hypothetical binding of cholesterol (*Ch*).

At high membrane loadings, **1Ch** exists in the membrane in two forms: the dispersed monomer (*D*) and the clustered form (*C*) (Fig. 4A). At the working temperature (30 °C), **1Ch** has a weak tendency to form clusters following an isodesmic, non -cooperative, aggregation process. The distribution between the *D* and *C* forms is determined by the clustering constant *K*_*DC*_ [24]:

(2) $$K_{DC}=\frac{\left[C\right]R_{L}}{\left[D\right]}$$

where *R*_*L*_ is the ratio of lipids over **1Ch**. The apparent rate constant, *k*_*1*_, can be written as a function of *K*_*DC*_ as:

(3) $$k_{1}=\frac{k_{D}R_{L}+k_{C}K_{DC}}{K_{DC}+R_{L}}$$

*k*_*D*_ is the rate constant of disaggregation of **1Ch**_**S**_ (that is, **1Ch** at the membrane-docked SAN) whereby only lipid molecules participate in the solvation of **1Ch** into the membrane, resulting in the formation of *D*. By contrast, *k*_*C*_ represents the rate constant for the disaggregation of **1Ch**_**S**_, where one or more membrane anchored **1Ch** participate in the solvation of the incoming **1Ch** molecule, leading to the formation of *C* (where *C* is any grouping of 2 or more molecules of **1Ch**) [24]. Given that **1Ch** does not translocate through the membrane in the timescale of these experiments, for calculations of *K*_*DC*_ we use the effective ratio of lipids over **1Ch**, *R*_*Le*_, which accounts for the fact that approximately half of the total lipids are located in the outer leaflet of the membrane [34], where **1Ch** is also located. *R*_*Le*_ is therefore estimated to be half the total lipid over **1Ch** ratio. Fitting of the changes in *k*_*1*_ with *R*_*Le*_ using eq. (3) returns a value for *K*_*DC*_ of 26 ± 11, which is about twice the value we have obtained from UV analysis of samples with increasing loading (Fig. 4A) [24,25]. This difference can be attributed to the following factors: (i) that equation (3) is a simplification of a more complex process, with *R*_*L*_ changing during the course of the experiment, (ii) that the effective *R*_*L*_ in the samples is, initially, much larger than calculated, due to the non-homogeneous distribution of the SANs amongst the vesicles. In addition, an increase of the L_o_ phase at large loads of **1Ch** would result on reduced lateral diffusion of membrane components, which may have an effect on the kinetic of SAN disassembly. From the fitting of the data, we obtain a *k*_*D*_ of 4.3 × 10^−4^ ± 3.0 × 10^−5^ s^−1^. The value of *k*_*C*_ obtained from the fitting is nearly an order of magnitude smaller, but the estimated number (e.g., 3.0 × 10^−5^ s^−1^) is subjected to a very large error and should therefore be taken with caution.

The second stage of **1Ch** incorporation into the lipid membrane, characterized by the transition from the **1Ch**_**I**_ intermediate form to the **1Ch**_**F**_ form, takes place with a rate constant *k*_*2*_ (Fig. 2D). There are, at least, two possible exchange mechanisms. **1Ch** may be exchanged between vesicles that collide with each other, or **1Ch** could dissociate from the membrane and travel to other vesicles as a monomeric specie in aqueous solution. The independence of *k*_*2*_ from vesicle concentration supports the latter mechanism. Additionally, the low but measurable solubility of monomeric **1Ch** in water (e.g., 11 nM, the CMC of the nanoparticle) further suggests this possibility [22]. Therefore, the exchange process likely involves the dissociation of **1Ch** from the membrane, followed by its incorporation into either the same membrane or another vesicle's membrane (Fig. 4B). Given the chemical structure of both **1Ch** and the lipid membrane, it is reasonable to assume that the second step—membrane anchoring following a collision—is very rapid, making dissociation from the membrane the rate-limiting step.

An approximate value for the binding affinity of **1Ch** to the membrane can be derived by assuming that the incorporation into the membrane is diffusion-limited. For a molecule of **1Ch**'s size and shape, the diffusion-limited rate constant can be estimated using a simplified form of the Smoluchowski equation (see the section “calculation of the diffusion limited rate constant” in the Supplementary Information for calculation details) [38]:

(4) $$k_{d}=4\pi D_{1Ch}R_{V}N$$

where *D*_*1Ch*_ is the translational diffusion coefficient for monomeric **1Ch**, *R*_*V*_ the hydrodynamic radius of the vesicle and *N* is Avogrado's number. The diffusion coefficient *D*_*1Ch*_ was calculated using the program Hydropro [39,40] and the hydrodynamic radius of the vesicle is the average radius of the vesicle determined by DLS [34]. The value of *k*_*d*_ obtained is 1.1 × 10^11^ M^−1^ s^−1^. The estimation of rate constants using this approximation is commonly applied in analyzing protein-protein and protein-ligand interactions. In such cases *k*_*d*_ represents a maximum value for *k*_*on*_, which must be adjusted to account for the fact that only collisions with the correct orientation result in the formation of the protein-protein or protein-ligand complex [41,42]. However, in our scenario, the binding of **1Ch** to the membrane is more appropriately viewed as a partitioning process between the aqueous phase and the membrane phase. Consequently, it is reasonable to assume that most collisions of **1Ch** with the lipid membrane interface result in the molecule anchoring, making *k*_*d*_ a good approximation for *k*_*on*_

The observed, macroscopic off-rate constant for **1Ch**-lipid binding is *k*_*2*_. Typically, when a molecule dissociates from surface-bound sites, it may re-bind to the surface, a phenomenon that can significantly impact the observed dissociation rate, especially in the case of macromolecules. Large molecules, due to their relatively small diffusion coefficients, are more likely to re-bind to the surface before escaping into the bulk solution [43]. In contrast, small molecules like **1Ch** have much larger diffusion coefficients. Combined with **1Ch**'s inherently low dissociation rate, it is reasonable to assume that the impact of re-binding on the observed rate is minimal and within the measurement's margin of error, making *k*_*2*_ a reliable approximation of *k*_*off*_ [43]. The value of *k*_*2*_ experiences a moderate decrease for membrane loadings above 1 % (*R*_*L*_ below 100). It has been shown that cholesterol binds more strongly L_o_ than L_α_ phases [44]. This increase may therefore reflect the fact that the *C* form of **1Ch**, more prevalent at larger membrane loadings, tends to accumulate within the L_o_ phase, from where the dissociation is less favored. To minimize the influence of the *C* form and the L_o_ phase, in our calculations we used as *k*_*2*_ value 2.4 × 10^−5^ s^−1^, which is the average of individual values of *k*_*2*_ calculated for membrane loadings at 1 % or below (*R*_*L*_ at or above 100). Notably, the *k*_*2*_ value calculated here is consistent with literature data on the rate of cholesterol desorption from unilamellar vesicles, further supporting our analysis [45]. Therefore, a binding constant can be calculated as the ratio of *k*_*on*_ and *k*_*off*_, as follows:

(5) $$K_{1Ch.L}=\frac{k_{on}}{k_{off}}=\frac{1.1\times 10^{11}\;M^{-1}s^{-1}}{2.4\times 10^{-5}\;s^{-1}}=4.5\times 10^{15}\;M^{-1}$$

**1Ch** has two distinct moieties, the porphyrin head group and the non-polar cholesterol tail. Considering these two moieties as two binding sites, we can write the binding constant of **1Ch** with the membrane, *K*_***1Ch****·L*_, as follows:

(6) $$K_{1Ch∙L}=K_{1∙L}K_{Ch∙L}{EM}_{i}$$

where *K*_***1****·L*_ and *K*_*Ch·L*_ are the binding affinities of the porphyrin and cholesterol moiety respectively and *EM*_*i*_ is the intramolecular effective molarity, a measure of the local concentration of these two moieties relative to each other. *EM*_*i*_ thus defined quantifies the entropic advantage of the binding of the second moiety taking place after the binding of the first moiety brings the binding partners (e.g., the membrane and the non-yet bound moiety) close to each other. In most molecular recognition events where *EM* is at play, it takes values that range from 0.1 to 10 M, the latter representing a high limit value in a solution where mid-size molecules are in contact in the liquid phase [31]. Since **1Ch** is in covalently attached to the cholesterol moiety we assume a high end effective molarity of 10 M. We have determined the binding affinity constant *K*_***1****·L*_, using **1**, chemically similar to the porphyrin domain of **1Ch**. An approximate value of *K*_*Ch·L*_ can therefore be calculated as:

$$K_{Ch∙L}=\frac{K_{1Ch∙L}}{K_{1∙L}{EM}_{i}}={1.6\times 10}^{11}\;M^{-1}$$

And, using the Gibbs equation we calculated the free energy of cholesterol binding, *ΔG*_*Ch*_, as −65 kJ mol^−1^. This value is remarkably consistent with those determined both experimentally and through computational methods for the binding of cholesterol to DMPC membranes [44,46]. This finding suggests that measurements of **1Ch** membrane dissociation can be used to determine the interaction energy of cholesterol with membranes of varying compositions. More importantly, it demonstrates that our analysis and the results derived from it are in line with previously reported data.

In summary, this study provides a detailed examination of the interaction and disassembly process of a self-assembled nanoparticle, i.e., a highly stable micelle, with a lipid membrane. This was achieved by taking advantage of the favorable spectroscopic properties of the porphyrin-cholesterol amphiphile **1Ch**. The spectroscopic changes observed are consistent with a two-step mechanism.

The first step involves the rapid, irreversible docking of the micelle to the membrane, driven by tight binding, which results from a multivalent effect where weakly-binding molecules work together to create a strong binding interface. Poisson distribution analysis of the data points to a scenario in which subsequent SAN docking tends to happen on lipid membranes containing already SAN, leading to the formation of SAN aggregates onto the lipid membrane. The subsequent disassembly of the nanoparticle within the membrane, prevents the nanoparticle from dissociating, thereby making the docking irreversible. Slower disassembly at high concentrations of **1Ch** in the membrane is consistent with the formation of **1Ch** clusters in these conditions. An increase of L_o_ domains at large **1Ch** loadings would also contribute to the slower disassembly, as the diffusion of lipids is slowed down within these domains.

In the second step, we observe the redistribution of **1Ch** across all available vesicle membranes. The slow rate of this phase is attributed to the gradual dissociation of **1Ch** from the membrane. We ruled out the possibility of translocation, or flip-flop, of **1Ch** within the lipid membrane as a mechanism for the observed in-membrane dilution due to the poor membrane permeability of its head group. By considering the likely diffusion-limited rate of association and analyzing the binding of a head group domain analogue, we were able to derive a value for the free energy of cholesterol binding consistent with literature values, supporting our proposed mechanism of self-assembled nanoparticle-membrane fusion and disassembly.

Finally, it is noteworthy that the self-assembled nanoparticle (SAN) described here, which demonstrates efficient membrane fusion and building block redistribution, along with previously reported features —such as tolerance to different metalloporphyrins, capacity for small molecule loading, and proven photosensitizing activity [23]— suggests significant potential for development in nanomedicine applications, including drug delivery and biosensing, a direction being pursued in our labs.

## CRediT authorship contribution statement

**Lilia Milanesi:** Writing – review & editing, Methodology, Investigation, Formal analysis, Data curation. **Salvador Tomas:** Writing – original draft, Validation, Supervision, Project administration, Methodology, Investigation, Funding acquisition, Formal analysis, Data curation, Conceptualization.

## Declaration of competing interest

The authors declare the following financial interests/personal relationships which may be considered as potential competing interests:Salvador Tomas reports financial support was provided by State Research Agency (Spain). Lilia Milanesi reports financial support was provided by State Research Agency (Spain). If there are other authors, they declare that they have no known competing financial interests or personal relationships that could have appeared to influence the work reported in this paper.
