# Supplementary material for: The interaction of a self-assembled nanoparticle and a lipid membrane: Binding, disassembly and re-distribution
Source: Heliyon. 2024 Oct 23;10(21):e39681. doi: 10.1016/j.heliyon.2024.e39681 (PMC11550047; doi:10.1016/j.heliyon.2024.e39681)
Supplement: Multimedia component 1 [file mmc1.docx]

**The interaction of a self-assembled nanoparticle and a lipid membrane: binding, disassembly and re-distribution**

***Lilia Milanesi and Salvador Tomas***

**General experimental methods**

Chemicals and solvents were obtained from commercial sources and used without further purification. UV-vis absorbance spectra were recorded with a ThermoFisher UV-1 Thermospectronic UV-vis spectrometer. The buffer used in this work was sodium phosphate buffer at pH 7.20, prepared by dissolving NaH_2_PO_4_ up to 10 mM in deionized water and the pH adjusted by addition of the necessary amount of a solution of NaOH 5M. All the experiments were carried at 30 ^o^C.

**Preparation of lipid vesicles.** A stock solution of lipids was prepared by dissolving dimyristoylphosphatidylcholine (DMPC) and cholesterol in a 8:2 molar ratio in ethanol, with a concentration of 20 mM A stock solution **1Ch** in ethanol with a concentration 1 mM was also prepared. To prepare the lipid vesicles, lipid and **1Ch** stocks were mixed in the appropriate proportions. The bulk of the solvent was removed under a nitrogen stream and traces of solvent removed under vacuum for a minimum of 1 hour. The solid film was then re-suspended in buffer by vortexing during 2 minutes. Excess of air and foam in the suspension were removed by sonicating the vortexed sample for 20 seconds in an ultrasound bath. The suspension was then extruded (Avanti Mini-Extruder) through 200 nm pore-size polycarbonate filters a minimum of 31 times.

**Experiments of micelle-vesicle incorporation.** In a typical experiment, a sample of vesicles with a concentration of lipids of 900 μM, was prepared according to the procedure detailed above. A sample of SAN of **1Ch** was generated by evaporation of the appropriate volume of the ethanolic stock of **1Ch** and re-suspension in buffer, up to a concentration of 3 μM of **1Ch**. The UV spectrum of this sample was recorded, after which it was combined with the suspension of lipid vesicles, resulting in a sample with a concentration of lipids 450 μM and of **1Ch** 1.5 μM. After mixing, the UV spectrum of the sample was recorded at regular intervals, up to 10 hours. This experiment was repeated with samples with concentration of lipids ranging from 11 to 750 μM.

**Re-distribution of 1Ch preloaded on vesicles**. In a typical experiment, a sample of vesicles containing **1Ch** was prepared according to the liposome preparation method detailed above, with a total concentration of **1Ch** 4 μM, and that of lipids 200 μM (*R_L_* = 50). The UV spectrum of this sample was recorded and then it was mixed with equal volume of a sample of lipid vesicles without **1Ch** ([lipids]=1800 μM). The concentrations in the resulting sample were 2 μM for **1Ch** and 1000 μM for the lipids. The UV spectrum of this sample was recorded at regular intervals, up to 10 hours.

**Membrane binding experiments**. In a typical experiment, 12 samples containing a constant concentration of **1** (2 μM) and increasing concentrations of lipid vesicles (ranging from 10 to 800 μM of lipid) in buffer were prepared. After preparation, the samples were left to equilibrate for a minimum of 30 minutes and their UV spectrum was recorded.

**Data analysis.**

**UV spectra**. The spectra of all the samples were recorded between 360 and 540 nm. In all the cases, the absorbance of the Soret band is located between 385 and 465 nm. In the rest of the acquired range **1Ch** does not absorb. Therefore, the data between 360-385 and 470-540 were used to re-construct the baseline and correct for the slope due to the light scattering of the liposomes. These data were fitted to a third polynomial function, and the resulting baseline was subtracted from the relevant spectra. The corrected spectra show only the contribution of species containing **1Ch** and were used for display purposes (Fig 2-3 -main text) and also for the determination of kinetic and binding constants (see below).

**Reconstruction of UV spectra at different ratios of lipid over 1Ch**. The theoretical spectra at any given **1Ch** loading can be reconstructed from the UV spectrum of the clustered *C* form and that of the dispersed *D* form. The spectra of the *C* and *D* form are already known [24].The clustering constant relates the concentration of *C* and *D* forms with the ratio of **1Ch** over lipids, *R_L_*, according to equation (2):

$K_{DC}=\frac{\left[ C \right]R_{L}}{\left[ D \right]}$ (2)

Eq. (2) can be re-arranged so that, the mol fraction of the *D* and *C* forms of **1Ch** can be determined from *R_L_* and the clustering constant as follows:

$x_{C}=\frac{K_{DC}}{K_{DC}-R_{L}}$ (S1)

$x_{D}=\frac{R_{L}}{K_{DC}-R_{L}}$ (S2)

The extinction coefficient at a given loading of **1Ch** and wavelength, ε, can determined from the extinction coefficients of C and D at that same wavelength, ε*_C_* and ε*_D_* as:

$\varepsilon=x_{C}\varepsilon_{C}+x_{D}\varepsilon_{D}$ (S3)

And substituting in eqs. (S1) and (S2) we have that

$\varepsilon=\frac{\varepsilon_{C}K_{DC}+\varepsilon_{D}R_{L}}{K_{DC}-R_{L}}$ (S4)

Equation (S4) allow to determine the extinction coefficient directly from *K_DC_* at any given *R_L_* and was used to reconstruct the spectra displayed in Supplementary Fig. S4 D.

**Fitting of the kinetic data**. The changes of the UV data (e.g., the corrected spectra of the Soret band region of **1Ch**) were fitted to a model that assumes two irreversible steps and that can be represented as:


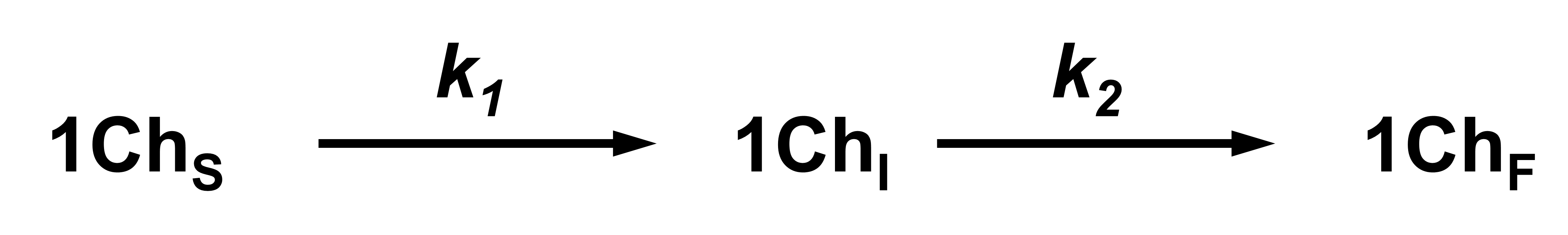


**Supplementary Scheme S1**

The data were fitted to this model using the program ReactLab KINETICS PRO (*JPlus Consulting*), from which the values of *k_1_* and *k_2_* (Supplementary Table S1), as well as the Soret band of each of the **1Ch** species (Fig. 2C, Supplementary Fig. S1) were obtained.


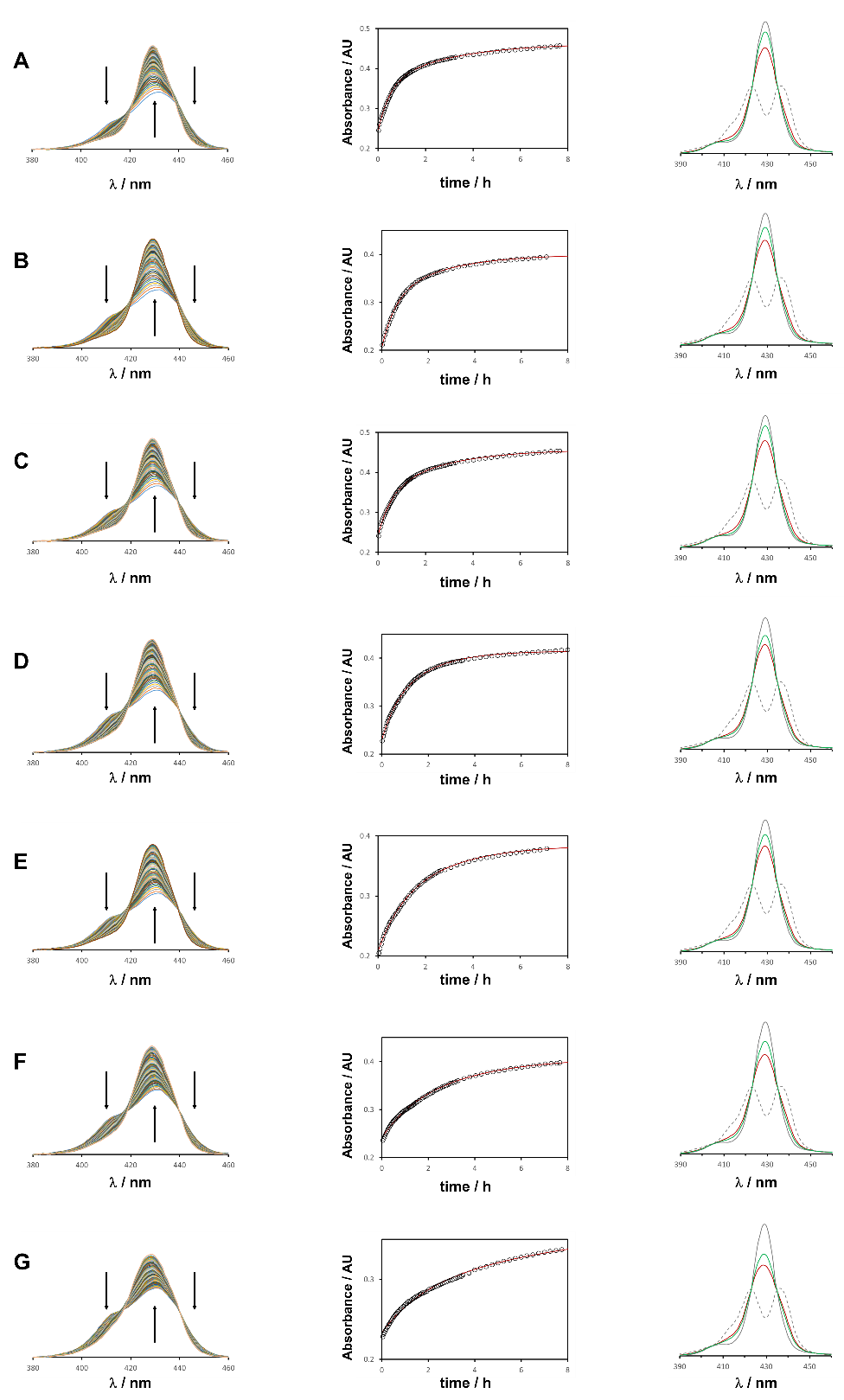


**Supplementary Fig. S1**. Left panels: changes in the Soret band of the spectrum of **1Ch** upon mixing with lipid vesicles. Middle panels: changes in absorbance at 428 nm (i.e., the wavelength where the change is the largest) with time (empty circles) and best fit to a two-step reaction model (Supplementary Scheme S1)(red line). Right panels: Soret band of the **1Ch_I_** (red trace) and **1Ch_F_** (green trace) species, extrapolated from the fitting of the data. The Soret band of pure membrane clustered and dispersed forms (*C* and *D* respectively, dashed and continuous grey lines) are also shown for comparison. These bands were obtained from the fitting of an in-membrane dilution experiment of **1Ch**.^[24]^ The concentration of **1Ch** was 1.5 μM in all experiments. The value of the molar ratio of lipids over **1Ch**, *R_L_*, was 510 (A), 175 (B), 105 (C), 60 (D), 35 (E), 20 (F) and 11 (G).

**Supplementary Table S1**. Rate constant for the first and second events of SANs-lipid vesicle interaction, at different concentration ratios of lipids and **1Ch**. The concentration of **1Ch** was 1.5 μM in all cases.

| [Lipids]/[**1Ch**] (% **1Ch**) | *k_1_* | *k_2_* |
| --- | --- | --- |
| 510 (0.20) | 3.7 x 10^-4^ ± 1.7 x 10^-4^ | 2.8 x 10^-5^ ± 8.1 x 10^-6^ |
| 300 (0.33) | 4.0 x 10^-4^ ± 2.0 x 10^-4^ | 2.4 x 10^-5^ ± 7.4 x 10^-6^ |
| 175 (0.57) | 3.0 x 10^-4^ ± 1.2 x 10^-4^ | 2.2 x 10^-5^ ± 4.6 x 10^-6^ |
| 105 (0.94) | 3.2 x 10^-4^ ± 8.9 x 10^-5^ | 2.4 x 10^-5^ ± 1.7 x 10^-6^ |
| 60 (1.7) | 2.4 x 10^-4^ ± 7.1 x 10^-5^ | 1.5 x 10^-5^ ± 3.3 x 10^-6^ |
| 35 (2.8) | 1.8 x 10^-4^ ± 4.4 x 10^-5^ | 1.6 x 10^-5^ ± 1.6 x 10^-6^ |
| 20 (5.0) | 1.4 x 10^-4^ ± 4.6 x 10^-5^ | 2.0 x 10^-5^ ± 7.8 x 10^-6^ |
| 11 (8) | 8.8 x 10^-5^ ± 3.9 x 10^-5^ | 1.2 x 10^-5^ ± 4.1 x 10^-6^ |

*k_1_* and *k_2_* are reported in s^-1^. The error of the measure of the rate constants is twice the standard deviation of two measures. The data are plotted in Fig 2E.

**Binding affinity of 1 for the lipid membrane**. The binding affinity of **1** for the lipid membrane was determined using a UV spectroscopy titration method, where the UV spectrum of **1** in buffer was recorded in solutions with an increasing concentration of lipids (10-800 μM). The experiments were designed such that the concentration of **1** was kept constant in all the samples. The observed spectrum for each sample was a combination of all the absorbing species, which included **1** free in solution, **1** associated with the membrane and the scattering of the liposomes. The absorbance due to the scattering of the lipids was removed as described in the data analysis section above (See ‘’UV spectra”). The program ReactLab EQUILIBRIA PRO (*JPlus Consultig*) was used to fit the corrected UV data to a model that assumes that **1** and the lipid molecules form a complex of 1 to 1 stoichiometry, with the apparent binding constant between the lipids and **1** , *K***_1_**_•_*_L_*, (Fig. 3A-B). The value of the binding constant obtained was:

$$K_{\boldsymbol{1}\cdot L}=2800\pm400 M^{-1}$$

The quoted error is twice the standard deviation of 2 measures.

***Structural parameters of 1Ch and 1Ch SAN***

To carry out the calculations for the in-membrane distribution it was necessary to estimate beforehand some structural parameter of **1Ch** and the nanoparticles (SAN). For the Poisson distribution we need a measure of the cross-section area of the porphyrin unit of **1Ch** (i.e., its head group), *A****_1Ch_***, and the radius of the SAN, *r_S_*.

***A measurement of A_1Ch_.*** The area occupied by the head group of **1Ch** was estimated from the crystallographic data of the structurally related Co metalloporphyrin **2** (Supplementary Figure S2) [33]. The molecular model of **1Ch** was generated using the program *Discovery Studio 2021* (BIOVIA) by simply replacing the metal centre by a Zn, remove one bipyridine ligand and replace the second one by a molecule of water. The cross section of the porphyrin is assumed to be the rectangular area capable of containing the totality of the porphyrin seen along the main plane of the ring, in the CPK representation. The area thus estimated is 2.0 nm^2^ (Supplementary Fig. S2).


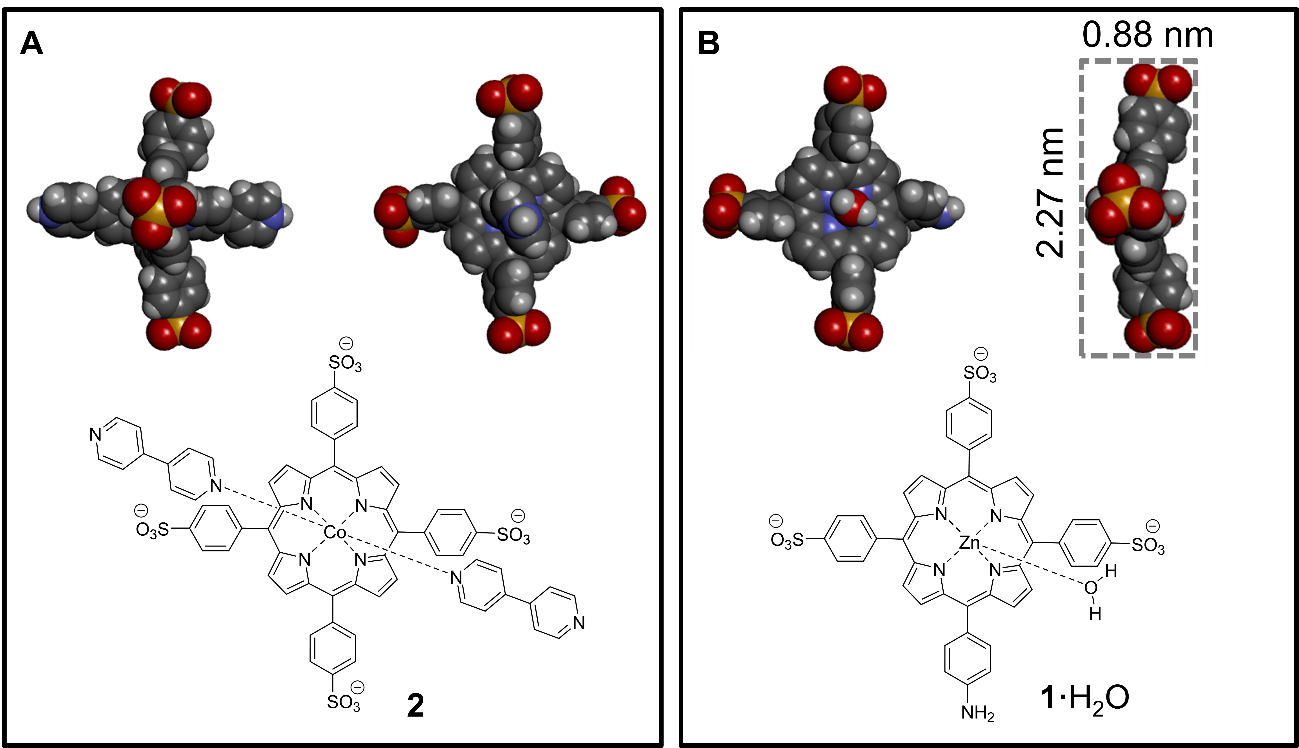


**Supplementary Fig. S2**. A. Chemical structure of metaloporphyrin 2 (bottom) and two orientations of the CPK model extracted from the crystal structure (top) [33]. B. Chemical structure of the complex **1**·H_2_O and two orientations of the CPK model. The side view of the porphyrin is shown circumscribed inside a rectangle of 2.0 nm^2^ (right).

***A measurement of r_S_.*** The radius of the SAN (*r_S_*) was estimated by measuring the apparent radius of the micelles in a representative EM image (Supplementary Figure S3). All the nanoparticles have a very similar radius, ranging between 5.5 and 4.5 nm, with an average of 4.4 nm.


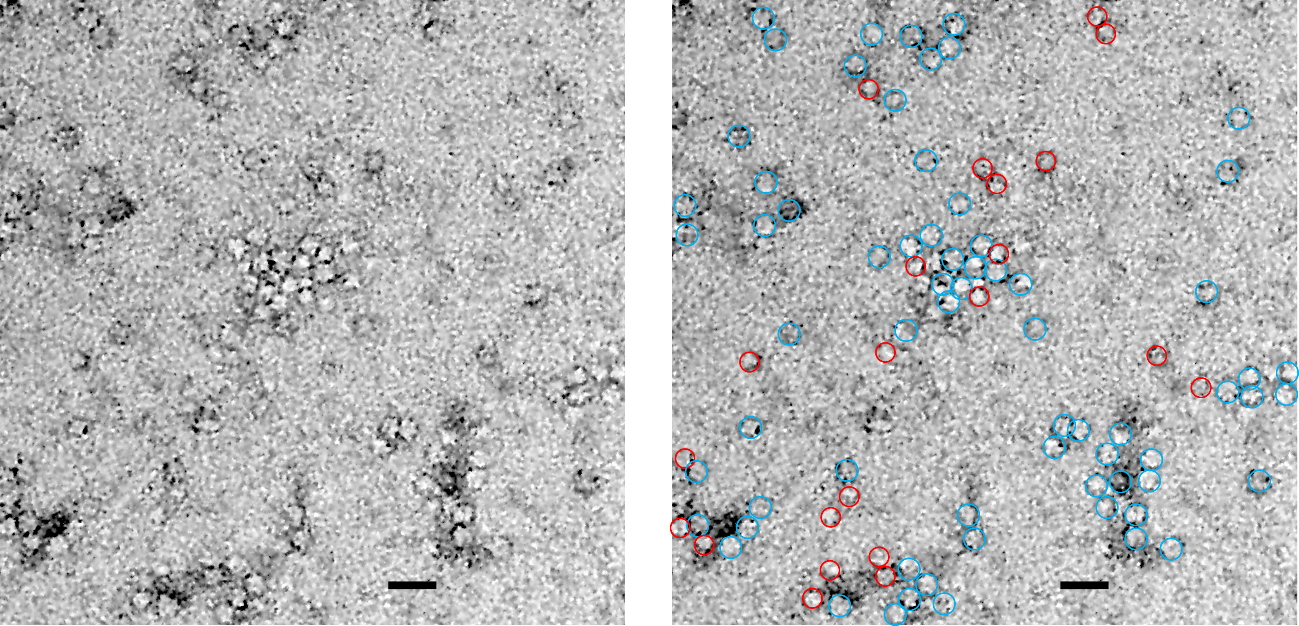


Supplementary Fig. 3S. A. EM image of **1Ch** SAN. B. Same image shown in A with circles of 9 (blue circles) and 8 (red circles) nm of diameter. There are 68 blue circles and 22 red circles in the image, which corresponds to a *r_S_* of 4.4 nm assuming spherical nanoparticles. Se ref. 22 for the experimental details on the preparation of the sample.

***Poisson distribution***

A Poisson distribution is a discrete probability distribution that expresses the probability of a given number of events occurring in a fixed interval (such as time, area or volume intervals, for example), given that these events occur with a known constant mean rate and are independent of each other. In our system, the interval is a given lipid vesicle and the event is the incorporation of a micelle (i.e., nanoparticle) of **1Ch** to the vesicle.

$P\left( k \right)=\frac{\lambda^{k}e^{-\lambda}}{k!}$ (S5)

Where *P*(*k*) is the probability that *k* events would take place and λ is the mean rate of events. In our system λ can be calculated as the ratio of the number of SAN, *n_S_*, over the number of vesicles *n_V_*, that is:

$\lambda=\frac{n_{S}}{n_{V}}$ (S6)

The number of vesicles per litre of sample can be calculated as the ratio of the number of molecules of lipid in the sample over the number of lipid molecules in a given vesicle, *n_Li_*_p_, that is:

$n_{V}=\frac{N_{a}[Lip]}{n_{Lip}}$ (S7)

Where *N_a_* is Avogadro´s number and the concentration of lipids is [*Lip*]. *n_Li_*_p_ can be estimated from the average radius of the vesicle, *r_v_* , and the area occupied by each lipid molecule *A_L_* as follows:

$n_{Lip}=\frac{8\pi{r_{v}}^{2}}{A_{L}}$ (S8)

The numerator of eq. (S8) is twice the total area of the membrane of the vesicle, because the membrane bilayer contains 2 leaflets of approximately the same surface.

The number of SAN per litre of sample, *n_S_* can be similarly determined, using here the number of **1Ch** molecules:

$n_{S}=\frac{N_{a}[\boldsymbol{1}\mathbf{Ch}]}{n_{\mathbf{1Ch}}}$ (S9)

Where the number of **1Ch** molecules per micelle, *n***_1Ch_** can be determined from the area of **1Ch**’s headgroup (*A***_1Ch_**) and the radius of the SAN, *r_S_* according to the following expression:

$n_{\mathbf{1Ch}}=\frac{4\pi{r_{S}}^{2}}{A_{\boldsymbol{1}\mathbf{Ch}}}$ (S10)

The value of all the parameters used in the calculation of the Poisson distribution can be seen in Supplementary Table S2 below.

Using these parameters as implemented in Eq. (S5) to (S10) allow us to simulate the apparent loading of **1Ch** on the vesicle membranes to be expected following **1Ch** incorporation and in the absence of re-distribution.

**Supplementary Table S2**. Parameters used in the simulation of the Poisson distribution.

| *A_L_* | 0.65 nm^2^ |
| --- | --- |
| *r_V_^a^* | 80 nm |
| [*Lip*] | 600 μM |
| *n_Lip_* | 3.87 x 10^5^ |
| *n_V_* | 9.34 x 10^11^ |
| *A***_1Ch_** | 2.0 nm^2^ |
| *r_S_* | 4.4 nm |
| [**1Ch**] | 2.0 μM |
| *n***_1Ch_** | 120 |
| *n_S_* | 1.00 x 10^13^ |

^a^ Value obtained from DLS measurements (see ref 34)


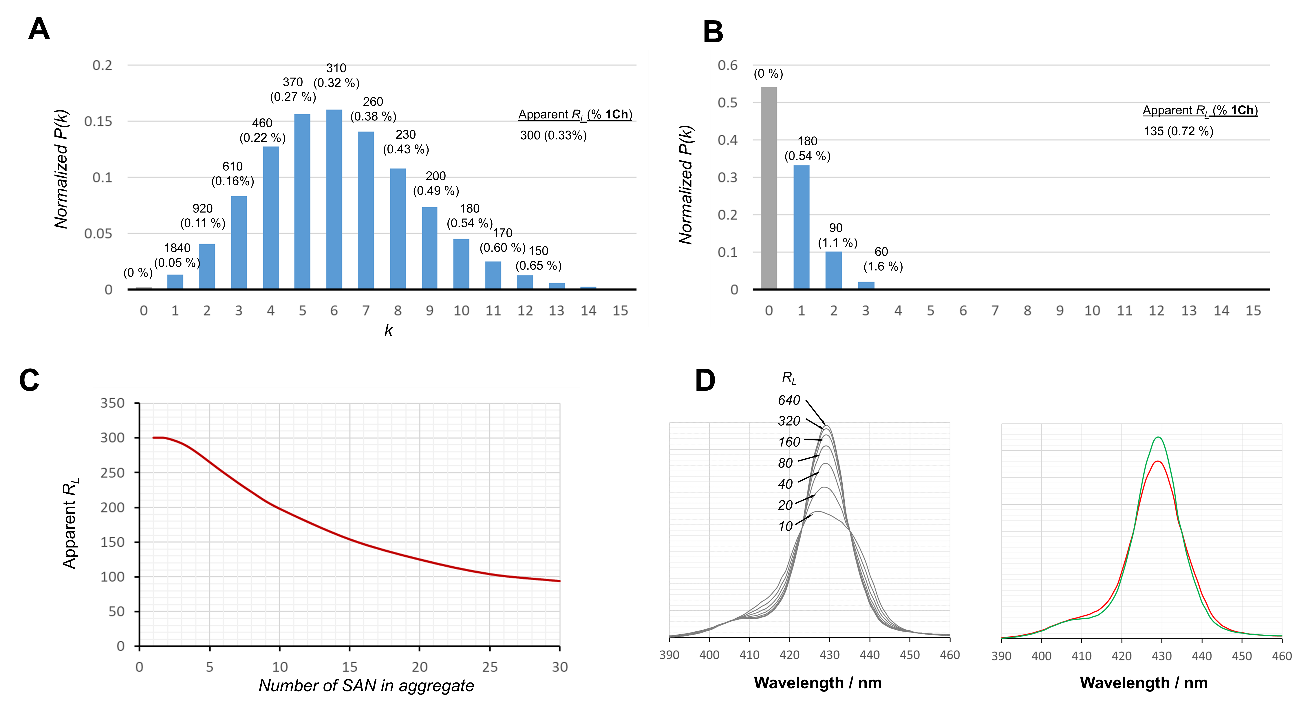


**Supplementary Fig. S4**. A. Graphical representation of the likelihood of a given vesicle (*P*) incorporating *k* number SAN according to a Poisson distribution, when each particle incorporates independently of each other. For each *k* number of nanoparticles incorporating the label on top of the bar shows the value of *R_L_* and % of **1Ch** that results from the incorporation. A homogeneous distribution would yield a *R_L_* of 300 corresponding to 0.33 % molar load of **1Ch** in the vesicle. B. Idem, when nanoparticles tend to aggregate in the surface of a vesicle forming aggregates of 20 SAN as an average. C. Changes of the apparent *R_L_* with the number of SAN in the aggregate. D. Reconstructed spectra of **1Ch** onto lipid membranes at different values of *R_L_* (left) and the spectra of species **1Ch_I_** (red trace) and **1Ch_F_** (green trace) obtained from fitting the data of an experiment with concentration of **1Ch** and lipids of 1.5 μM and 450 μM respectively (i.e., *R_L_* = 300).

***Calculation of the diffusion limited rate constant***

The diffusion limited rate constant can be estimated using the Smoluchowski equation [38]:

$k_{d}=4\pi(D_{1Ch}+D_{V})(r_{1Ch}+r_{V})N$ (S11)

Were *D_1Ch_* and *D_V_* are the translational diffusion coefficients for **1Ch** and the lipid vesicles respectively, *r_1Ch_* and *r_V_* their respective hydrodynamic radius and *N* is Avogrado’s number. In our system, we have that the vesicle is much larger than monomeric **1Ch**. The implications are that

$D_{1Ch}\gg D_{V}$ (S12)

$r_{V}\gg r_{1Ch}$ (S13)

Which allow us to simplify the expression, obtaining equation 4

$k_{d}=4\pi D_{1Ch}r_{V}N$ (4)

The average vesicle radius for our samples is 80 nm, according to DLS measurements [34]. The diffusion coefficient of **1Ch** was determined using the program HYDROPRO [39], using as input a molecular model of **1Ch**. In turn, this molecular model of **1Ch** was obtained by minimizing the structure of the metal-free version of **1Ch** using the AMBER force field as implemented in Macromodel 4.0 [40]. The diffusion coefficient obtained for **1Ch** is 1.84 x 10^-6^ cm^2^s^-1^.
